# Interference between Conscious and Unconscious Facial Expression Information

**DOI:** 10.1371/journal.pone.0105156

**Published:** 2014-08-27

**Authors:** Xing Ye, Sheng He, Ying Hu, Yong Qiang Yu, Kai Wang

**Affiliations:** 1 Department of Neurology, The First Affiliated Hospital of AnHui Medical University, Hefei, Anhui Province, China; 2 Department of Radiology, The First Affiliated Hospital of AnHui Medical University, Hefei, Anhui Province, China; 3 Department of Psychology, University of Minnesota, Minneapolis, Minnesota, United States of America; 4 State Key Laboratory of Brain and Cognitive Science, Institute of Biophysics, Chinese Academy of Sciences, Beijing, China; University G. d'Annunzio, Italy

## Abstract

There is ample evidence to show that many types of visual information, including emotional information, could be processed in the absence of visual awareness. For example, it has been shown that masked subliminal facial expressions can induce priming and adaptation effects. However, stimulus made invisible in different ways could be processed to different extent and have differential effects. In this study, we adopted a flanker type behavioral method to investigate whether a flanker rendered invisible through Continuous Flash Suppression (CFS) could induce a congruency effect on the discrimination of a visible target. Specifically, during the experiment, participants judged the expression (either happy or fearful) of a visible face in the presence of a nearby invisible face (with happy or fearful expression). Results show that participants were slower and less accurate in discriminating the expression of the visible face when the expression of the invisible flanker face was incongruent. Thus, facial expression information rendered invisible with CFS and presented a different spatial location could enhance or interfere with consciously processed facial expression information.

## Introduction

Over the past several decades, experimental psychologists have demonstrated that a remarkably wide range of visual information processing can occur outside of awareness, which in turn significantly influence various aspects of human behavior [1]–[3]. In particular, it has been shown that emotional information (e.g., fearful, disgust, or happy expressions) from the face or the body can be processed in the absence of visual awareness [4]. Neuroimaging studies using backward masking [5], [6], binocular rivalry [7]–[9] or Continuous Flash Suppression [10], [11] have demonstrated that the haemodynamic responses of the amygdala, superior temporal sulcus face sensitive area, and anterior cingulate can be elicited by emotional stimuli rendered invisible.

Additionally, emotional stimuli can modulate ongoing cognitive processes [12], [13]. Subliminal affective responses to stimuli can be elicited and implicitly affect our perception of certain context, influencing our choices in approach-avoidance behaviors [14]–[17]. A number of studies using subliminal affective priming stimuli have shown the influence by subliminal presented prime on response toward supraliminal target including pleasant and unpleasant words [18], [19], happy and sad/angry facial expression [14], [16], [17], [20] and positive/ negative pictures [21], [22]. Also, exposure to subliminal prime with words related to thirst increased participants' beverages consumption [15].

Binocular rivalry, the alternating percept between two eyes' images when different images are presented dichoptically, can be used to study unconscious visual information processing. Previous fMRI studies demonstrate that during binocular rivalry suppressed emotional faces can be processed and generate stronger amygdala activity compared with neutral stimuli [8], [9]. A relatively new procedure modified from binocular rivalry called Continuous Flash Suppression (CFS) is a more controlled way to render visually presented stimuli invisible [23], [24]. By exposing one eye to continuously flashing contour-rich and high-contrast random patterns, CFS makes a visual stimulus invisible for a relatively long period of time [23], and has become a very effective technique for investigating the mechanisms of unconscious visual information processing. With faces suppressed by CFS, responses to suppressed fearful faces remain robust in the amygdala and the superior temporal sulcus [10]. In behavioral studies, when initially suppressed, upright faces broke interocular suppression faster than inverted faces [25], and fearful faces faster than neutral faces [26]–[28]. A number of recent studies indicate that the exposure to an emotional stimulus rendered invisible under CFS can evoke adaptation effects and influence the consequent reaction [29], [30]. However, some have questioned whether these effects could be attributed to the emotional level analysis or simply due to the image features that make stimuli more like faces [31].

Previous studies have shown that unconsciously processed expression information could modulate the processing of subsequently presented images [29], [30]. The study by Jiang et al. shows that a back-ward masked emotional face could prime and interfere with affective judgment of a subsequently presented visible face, demonstrating that the emotion conflict could be trigged by backward-masked emotional information [32]. Given that back-ward masking and CFS could have different consequences [33], in this study, we investigated the interaction between consciously perceived and unconsciously processed emotional information, using CFS to render images invisible. The current study focused on the potential influence of an invisible facial expression on the expression judgment of a spatially non-overlapping visible emotional face. If the unconscious expression information processing could influence the judgment of consciously perceived facial expression, then we would expect to see a congruency effect - the reaction time would be shorter and accuracy higher when the expressions of the visible and invisible faces are incongruent than when they are congruent. Thus we investigated this issue by presenting a target face visibly on one side of the fixation and presenting another face on the other side of the fixation, but rendered invisible using CFS. The results of our study show a robust congruency effect, suggesting that unconsciously processed information could enhance or interfere with consciously processed information, even when they are spatially non-overlapping.

## Methods

### Participants

Twenty-eight participants, (22.3±1.82 years old, 12 male) right-handed, healthy native Chinese-speaking students at Anhui Medical University, participated in this experiment. All participants were naive to the purpose of the experiment. All had normal or corrected to normal visual acuity, and no history of neurological trauma or psychiatric disorders. All participants were paid for their participation and gave written informed consent in accordance with the procedures and protocols approved by the Human Participants Review Committee of Anhui Medical University.

### Stimuli

Photographs of four male and four female faces taken from 8 Chinese actors were assembled for use in the present experiment. Each face was photographed against a gray background in full frontal view (no glasses, jewelry, or paraphernalia). Each of these 8 faces was photographed twice, once with happiness expression and once with fear expression, for a total of 16 photographs. The individuals reported in this manuscript have given written informed consent (as outlined in PLOS consent form) to publish these case details.

Stimuli were generated with MATLAB (www.mathworks.com) and presented on a Lenovo 19-inch LCD monitor (set at 1,440*900 pixel resolution and a refresh rate of 60 Hz) using the psychophysical toolbox (www.psychtoolbox.org; Brainard, 1997). The left and right eye's images were displayed side-by-side on the monitor and viewed through a mirror stereoscope mounted in front of a chinrest. A frame (12.4° *12.4°) that extended beyond the outer border of the stimulus and fixation point was presented to facilitate stable convergence of the two eyes' images. The viewing distance was 43.5 cm.

### Procedure

Stimuli were displayed against a gray background. During the whole experiment two black-and-white frames (12.4° *12.4°) were presented side by side on the screen, such that one frame was visible to each eye. In the center of each frame a red central fixation cross (0.66° *0.66°) was displayed. Participants were asked to maintain stable fixation throughout each experiment block. In all experiments, face stimuli were 8 happy face photographs (four female, subtended 3.3° * 4.0° of visual angle) and 8 fearful face photographs (four female, with the same visual angle as happy faces).

**Figure 1:**
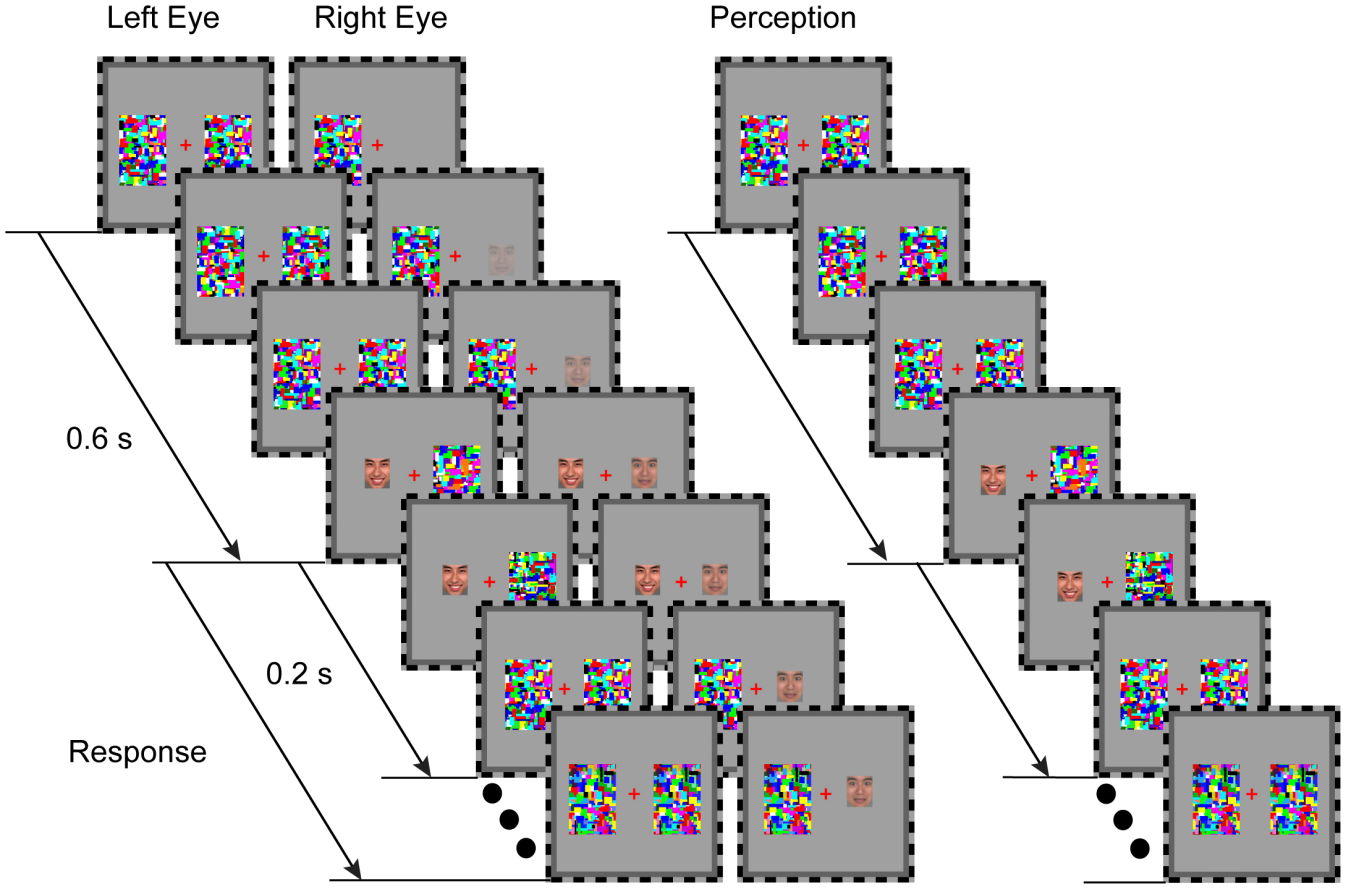
Schematics depiction of an example trial with incongruent facial expression. The suppressed face image was presented to one eye with its contrast gradually ramped up, and was expected to be suppressed by the dynamic noise. The contrast of the suppressed face image was ramped up linearly from 0 to 40% of its original contrast within a period of 600 ms from the beginning of the trial and then remained constant until the participant had made a response. When the suppressed face reached a stable contrast, the target face image (happy or fearful) was presented on the other side of the fixation cross to both eyes, thus visibly for 200 ms before it disappeared. Participants were asked to respond, as quickly as possible, to the target face image, by pressing one of two keys to indicate whether it was happy or fearful.

The main experiment was divided into two phases: a block of the training phase followed by three blocks of the test phase. Each trial started with a 1.5 s presentation of the fixation cross and the black-and-white frame only. Then a pair of colored, high-contrast, Mondrian-like CFS masks (similar to those used by Jiang et al. [25] and Sterzer et al. [34]) measuring 3.3°*4.6° were flashed to the participant's dominant eye (determined as the eye with longer dominance time based on a quick binocular rivalry test using gratings) at a frequency of 15 Hz, while a happy or fearful face (distractor stimulus) subtending 3.2° * 4.0° of visual angle was introduced to the nondominant eye (see Figure 1, The horizontal distance between the centers of this pair of masks was 6.6°). The face was presented either to the left or to the right of the fixation cross, at a random location within the area corresponding to the location of the CFS masks. The contrast of the suppressed face stimulus was ramped up linearly from 0 to 40% of its original contrast within a period of 600 ms from the beginning of the trial and then remained constant until the participant had made a response. At the time when the suppressed face reached the stable contrast (following 600 ms of contrast ramping), a target face image (happy or fearful) was presented on the other side of the fixation cross, binocularly, thus visibly. The target face was presented for 200 ms before it disappeared. The participants were asked to decide whether the face was happy or fearful as quickly and accurately as possible by pressing one of two buttons. Reaction time was calculated as the time from the onset of the target face to the time of the button press. Participants were asked to press another key in each trial if they saw any part of the suppressed face (failure of suppression), and such trials were excluded from further analysis.

The order of the faces was pseudo-randomized so that no more than four faces with the same affective valence were presented consecutively. The three test blocks each consisted of 80 trials. Participants took 3 min rest before starting the next block.

## Results

**Figure 2:**
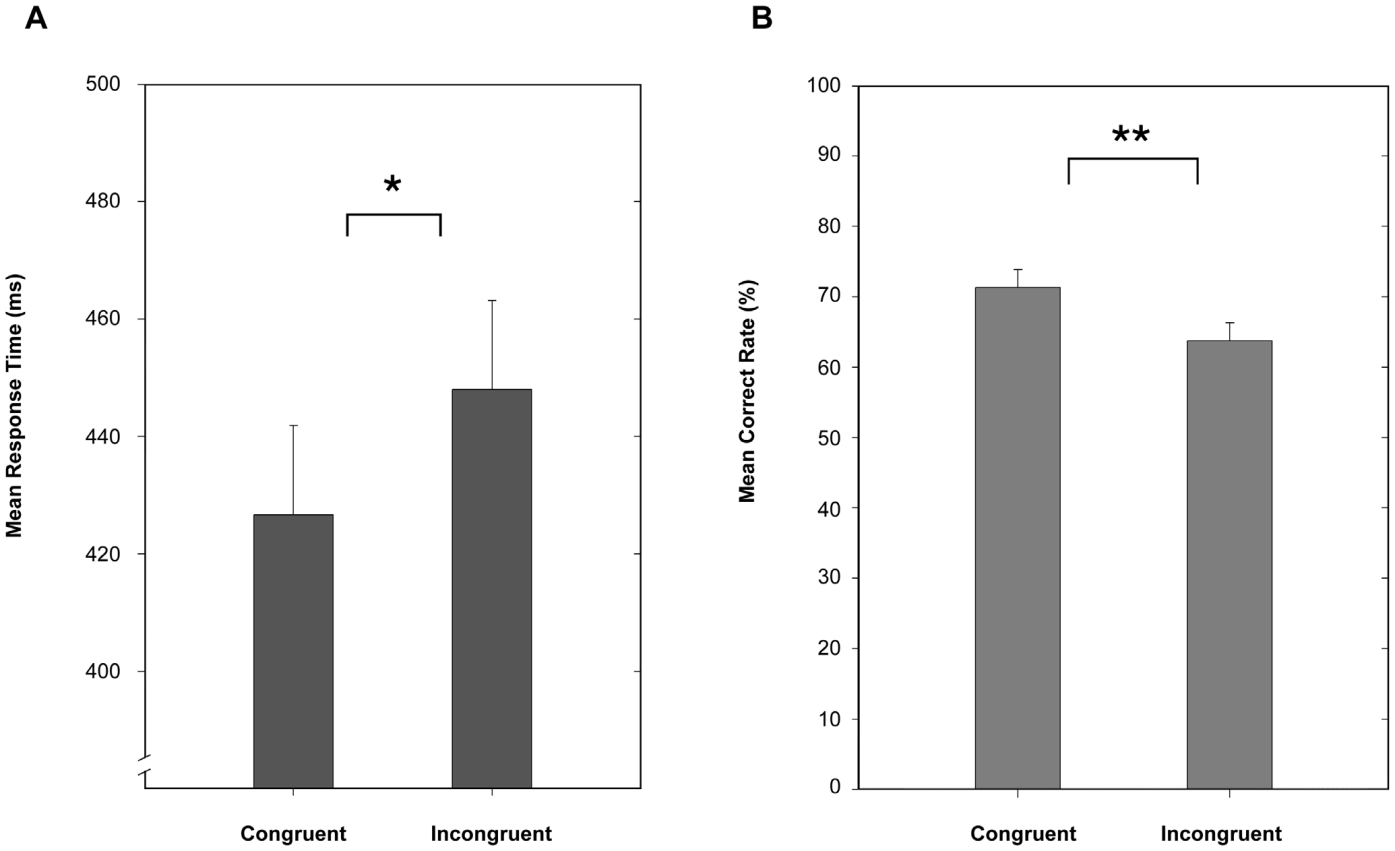
Summarized results of the main experiment. A. mean reaction times in the congruent and incongruent conditions, showing that the reaction time is significantly slower in the incongruent than that in the congruent condition (448 ms vs. 427 ms, t(27) = 3.13, p<.005); B. mean accuracy for the congruent and incongruent conditions, showing that participants responded more accurately in the congruent than in the incongruent conditions (71.31% vs. 63.73%, t(27) = 2.32, p<.005).

The expressions (happy/fearful) depicted in the subliminally presented stimulus and in the visible target were either congruent or incongruent. We analyzed the data based on the congruency of the expressions. Results showed that a vast majority of the 28 participants had shorter response time for the congruent than the incongruent condition. Reaction times were calculated from the correct trials only. The average response time in the congruent condition was significantly shorter than that in the incongruent condition (427 ms vs. 448 ms), t(27) = 3.13, p<.005 (Fig. 2A). In addition, participants were more accurate judging the expression of the target face when the invisible distractor had a congruent than incongruent expression (71.31% vs. 63.73%), t(27) = 2.32, p<.005 (Fig.2B). In addition to the independent objective measure of the suppression effectiveness (described below), we also had an indication of the effective suppression from participants' reports during the main experiment, since the flanker face broke suppression in only 1.5% of trails. These results demonstrate that unconsciously presented emotional information at one location influenced the judgment of consciously processed expression information at a different spatial location.

### Objective Measures of the Suppression Effectiveness

**Figure 3:**
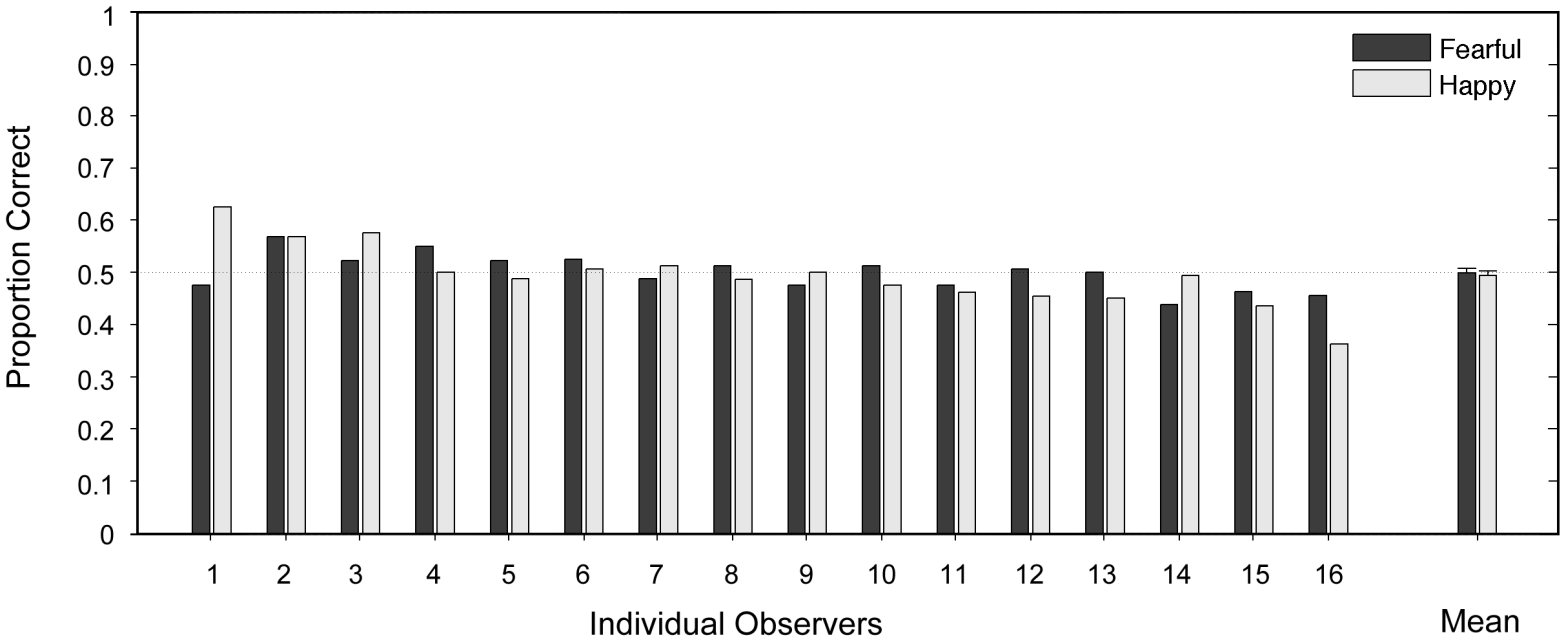
Results of the suppression effectiveness control experiment. Proportions of correct responses for 16 participants in a two-alternative forced choice task in determining on which side a face image was presented while the face image was suppressed in the same way as in the main experiment. None of the participants performed significantly different from chance, as demonstrated by binominal tests (p>0.05 for all 16 participants). Error bars in the mean performance are 95% confidence intervals.

Because of the importance of keeping the suppressed image invisible during the test phase, in addition to asking the participants to report during the congruency experiment if the suppressed image became visible (including partially visible), we also performed an objective measure of suppression effectiveness following the main experiment. Sixteen of the participants (8 male, 8 female) completed a two-alternative forced choice (2AFC) task. The stimulus parameters in this 2AFC experiment were the same as those in the main experiment. Because in the main experiment the suppressed stimuli disappeared when the participant made a judgment about the target and pressed the key, we set the presenting duration for the suppressed stimuli in the 2AFC experiment according to the mean response time from the main experiment. During each trial, participants were asked to make a forced choice response indicating on which side of the fixation the face image was presented. The results showed that none of the participants performed significantly different from chance as demonstrated by binominal tests (p>0.05 for all 16 participants), with a mean correct percentage of 0.496±0.010 (mean ±SEM, t(15) = 0.3454, p>0.7). Thus the 2AFC objective test provided additional assurance that participants were not aware of the suppressed stimuli (see Figure 3).

The above control experiment indicated that participants were not aware of which side the suppressed face was presented. In another set of 16 participants, we further assessed whether participants were able to make judgments about the valence of the suppressed face. During each trial, participants were asked to make a forced choice response indicating on whether the face was happy or fearful by pressing one of two buttons. The results showed that none of the participants performed significantly different from chance, as demonstrated by binominal tests (p>0.05 for all 16 participants) with a mean correct percentage of 0.4858±0.0079(mean±SEM, t(15) = 1.793, p>0.05). Thus results from the two control experiments give us high confidence that participants did not perceive the suppressed face nor did they have an explicit representation of the valence of the suppressed face.

Since the objective awareness experiment (location judgment) was run on 16 out of the participants in the main experiment, and there is the possibility that the congruency effect observed in the main effect was primarily due to the trials in which the suppressed face was presented for longer durations than the average duration, we further checked the congruency effect in the main experiment only using data from these 16 participants and only using trials with duration that are equal to or shorter than the trials in the awareness check. With this subset of data meeting the above criteria, the average response time in the congruent condition was still significantly shorter than that in the incongruent condition (381 ms vs. 396 ms, t(15) = 2.70, p<0.05); and the accuracy in the congruent condition also remained higher than that in the incongruent condition (67.46% vs. 60.02%, t(15) = 2.58, p<0.05).

## General Discussion

The present study was designed to investigate whether facial expression from an invisibly presented flanker could influence the recognition of expression of a visible target face. The flanker stimuli were rendered invisible using continuous flash suppression [23], [24]. Results showed that the exposure to subliminal facial expressions presented at different spatial location indeed influenced the expression recognition of the visible face, as seen in the congruency effect: congruent facial expressions facilitated participants' responses both in terms of the faster reaction time and improved accuracy. This observed congruency effect is consistent with the many published reports of unconscious processing of emotional information [15], [17], [29], [32]. Since the happy and fearful faces have systematic image differences (e.g., sizes of eyes and mouths, etc), we acknowledge the possibility that the observed congruency effect could be attributed to the congruency between the different images.

Early studies have shown that invisible affective information could be processed in the brain [35], [36]. Invisible affective stimuli can generate a subliminal affective priming effect that influences subsequent emotional processing [17]. Results from the present study using CFS to render faces invisible are consistent with and extends finding using back-masked faces [32] in showing that subliminal affective facial expression could induce emotional conflict on a visibly presented spatially non-overlapping face.

Several imaging studies investigating the emotional information processing under CFS have shown that the subliminal emotional stimuli during binocular rivalry suppression also could be processed especially in the amygdala [8]–[10]. One possibility is that the suppressed emotional information could engage amygdala via the subcortical pathway through the superior colliculus and the pulvinar [9], [37], [38]. However, there is still a debate on the role of amygdala in the detection of fear signal [8], [26]–[28], [37], in particular with one study showing that the advantage of fear detection still exists even when bilateral amygdala were damaged [28].

The congruency effect observed here in expression judgment reflects a conflict resolution process. Congruency effects between facial expressions (fearful/happy) with emotional words (e.g., “happy” or “fear”) written on faces have been reported before [39], but what is different here is that the conflicting expression information is not between two consciously perceived inputs, but between a visible target and an invisible flanker. The interaction between conscious and unconscious emotional information could potentially occur in the amygdala, or at high-level cortical sites. Further neuroimaging research will be needed to investigate the specific neural mechanisms responsible for the integration of conscious and unconscious input information.

## Conclusions

The present study demonstrated that unconsciously processed facial expression information can influence the recognition of consciously processed facial expression information, across different spatial locations. Our results add to the growing evidence that invisible information can be processed rapidly and can directly influence conscious behavior.
